# Integrated gene-free potato genome editing using transient transcription activator-like effector nucleases and regeneration-promoting gene expression by *Agrobacterium* infection

**DOI:** 10.5511/plantbiotechnology.23.0530a

**Published:** 2023-09-25

**Authors:** Naoyuki Umemoto, Shuhei Yasumoto, Muneo Yamazaki, Kenji Asano, Kotaro Akai, Hyoung Jae Lee, Ryota Akiyama, Masaharu Mizutani, Yozo Nagira, Kazuki Saito, Toshiya Muranaka

**Affiliations:** 1 RIKEN Center for Sustainable Resource Science, Kanagawa 230-0045, Japan; 2 Graduate School of Engineering, Osaka University, Osaka 565-0871, Japan; 3 National Institute of Agrobiological Sciences, National Agriculture and Food Research Organization, Ibaraki 305-8518, Japan; 4 National Agricultural Research Center for Hokkaido Region, National Agriculture and Food Research Organization, Hokkaido 082-0081, Japan; 5 Graduate School of Agricultural Science, Kobe University, Hyogo 657-8501, Japan; 6 Agri-Bio Research Center, Kaneka Co., Shizuoka 438-0802, Japan

**Keywords:** genome editing, regeneration-promoting gene, steroidal glycoalkaloids, TALEN, transient gene expression

## Abstract

Genome editing is highly useful for crop improvement. The method of expressing genome-editing enzymes using a transient expression system in *Agrobacterium*, called agrobacterial mutagenesis, is a shortcut used in genome-editing technology to improve elite varieties of vegetatively propagated crops, including potato. However, with this method, edited individuals cannot be selected. The transient expression of regeneration-promoting genes can result in shoot regeneration from plantlets, while the constitutive expression of most regeneration-promoting genes does not result in normally regenerated shoots. Here, we report that we could obtain genome-edited potatoes by positive selection. These regenerated shoots were obtained via a method that combined a regeneration-promoting gene with the transient expression of a genome-editing enzyme gene. Moreover, we confirmed that the genome-edited potatoes obtained using this method did not contain the sequence of the binary vector used in *Agrobacterium*. Our data have been submitted to the Japanese regulatory authority, the Ministry of Education, Culture, Sports, Science and Technology (MEXT), and we are in the process of conducting field tests for further research on these potatoes. Our work presents a powerful method for regarding regeneration and acquisition of genome-edited crops through transient expression of regeneration-promoting gene.

## Introduction

Genome-editing technologies can introduce a mutation at a specific site via a target gene and a genome-editing enzyme. Major classes of genome-editing enzymes include zinc-finger nucleases (ZFNs), transcription activator-like effector nucleases (TALENs), and clustered regularly interspaced short palindromic repeats-associated protein 9 (CRISPR-Cas9), and each has undergone significant development as a gene-editing framework (Voytas 2013; Voytas and Gao 2014).

Conventionally, to construct a plant with a target mutation, molecular breeders have used the Targeting Induced Local Lesions in Genomes method, which involves selecting desirable mutants in which a target gene is destroyed from a pool of mutants with random mutations caused by radiation or mutagenic chemicals. However, genome-editing technologies have enabled obtaining target mutants quicker than that from conventional methods and preventing mutations in genes other than the target gene. Because plants obtained by genome-editing technologies cannot be distinguished from mutants obtained using the conventional methods, such plants may not be regulated as strictly as genetically modified plants. Given these advantages, genome-editing technologies are gaining increasing attention as novel plant breeding technologies. Moreover, the Japanese government has created new regulations for handling genome-edited organisms, with the main principle being that genome-edited organisms must be free of foreign genes.

In animals, a genome-edited mutant can be easily obtained by introducing a genome-editing enzyme in the form of an RNA, protein, or complex into a fertilized egg without incorporating the gene of the genome-editing enzyme into the genome. In contrast, obtaining a fertilized ovum in plants is difficult; therefore, a genome-editing enzyme is introduced as an RNA, protein, or complex into an isolated cell or protoplast. As plants are pluripotent, an individual can therefore be regenerated from the manipulated cell or protoplast to successfully obtain a genome-edited individual. Moreover, these individuals do not contain the gene of the genome-editing enzyme in their genome. However, few species and cultivars can be regenerated from protoplasts, and culture mutations are very often generated over the course of regeneration from protoplasts; these problems can limit the range of applications of this approach (Fossi et al. 2019).

For crops that reproduce via seed, a multi-step process can be used to obtain a desirable mutant in which the gene of the genome-editing enzyme is not incorporated into the genome. This is done by removing the gene through crossing and genetic segregation after incorporating the gene of the genome-editing enzyme into the genome. However, for crops such as potato, whose elite cultivars are maintained via vegetative propagation, once hybridized with the constructed elite cultivar, the crop becomes a different cultivar from the original. Because of this propagation method, the methods for removing the gene of the genome-editing enzyme that is incorporated during crossing are useful for creating a mother plant for breeding, but does not directly improve varieties. Thus, there is demand for a novel method of obtaining a genome-edited plant in which a mutation can be introduced into a specific gene without an exogenous gene also being incorporated into the genome. Therefore, we propose a method of plant genome editing by transient site-specific nuclease expression via *Agrobacterium*, a process that has been called agrobacterial mutagenesis (Yasumoto et al. 2020). *Agrobacterium tumefaciens* harboring a site-specific nuclease expression vector was used to infect potato stems. Subsequently, we checked that shoots regenerated on a medium containing phytohormones without selection reagents and reported that *Agrobacterium* transient expression resulted in shoots that contained genome-edited cell lines.

We then developed a method of using regeneration-promoting genes to improve our original framework. The *ipt* gene from Agrobacterium contributes to plant hormone biosynthesis. Temporarily activated *ipt* promotes shoot formation in plant tissue, whereas constitutively expressed *ipt* causes morphological abnormalities. Previously, a marker-free method of genetic modification called the MAT vector method has been developed based on this phenomenon (Ebinuma et al. 1997). Moreover, many genes are known to promote regeneration when transiently expressed, but also to develop calli and/or other morphological abnormalities when constitutively expressed. For example, the *BBM* (Baby Boom) genes of *Arabidopsis thaliana* and *Brassica napus* enhance regeneration ability in tobacco (Srinivasan et al. 2007), promote transformation in chili pepper (Heidmann and Boutilier 2015), and enhance transformation in monocots (Lowe et al. 2016). In addition, *ESR1* and *ESR2* of *Arabidopsis thaliana* promote shoot regeneration (Banno et al. 2001; Ikeda et al. 2006), and the ectopic, postembryonic expression of *Arabidopsis* *LEC2* in transgenic plants induces the formation of organ-like structures and often confers embryonic characteristics to seedlings (Stone et al. 2001). Furthermore, induced overexpression of the *Arabidopsis* *WUS* gene causes high-frequency somatic embryo formation in all tissues (Zuo et al. 2002). Thus, we hypothesized that the simultaneous transient expression of these genes and site-directed restriction enzymes would allow the regeneration of shoots from potato stem fragments, thereby allowing us to obtain genome-edited mutants.

## Materials and methods

### Plant materials

Murashige and Skoog (MS) medium containing 3% sucrose solidified with 0.8% agar was used for in vitro cultivation of *Solanum tuberosum* cv. Sassy, Sayaka and May Queen.

### Construction of vectors containing regeneration-promoting gene

*Ipt* gene was synthesized based on agrobacterium A281 sequence (ACCESSION X17432). A plant transformation vector pSuehiro108 was prepared by binding the 35S RNA promoter of cauliflower mosaic virus (35SP), the 5′ untranslated sequence (ADH5′) of *Arabidopsis thaliana*, platinum Gate TALEN targeting the *sterol side chain reductase 2* (*SSR2*) gene (Yasumoto et al. 2019), and the *Arabidopsis* *HSP* gene terminator (HSP-T) in the forward direction, and the kanamycin selection marker gene (Km resistance) and the *ipt* gene fragment in the opposite direction, utilizing restriction enzyme sites set in the opposite ends, based on the binary vector pKT19 (Umemoto et al. 2016). Note that as the target sequence of *SSR2*, the same region as that described in a literature (Sawai et al. 2014) was utilized. At the same time, a vector pSuehiro105 that did not contain the *ipt* gene was prepared (Supplementary Figure S1). *BBM*, *ESR1*, *ESR2*, *LEC2*, and *WUS* genes was cloned from cDNA or genome DNA of *Arabidopsis thaliana*. Each gene was expressed by 35SP, ADH5′, and HSP-T (Supplementary Figure S1).

### *Agrobacterium* infection of potato stems

The *Agrobacterium tumefaciens* GV3101 pMP90 strain was transformed with the above vectors. Transformed *Agrobacterium* clones were selected on LB medium (5 g l^−1^ yeast extract, 10 g l^−1^ tryptone, 10 g l^−1^ NaCl) containing 50 mg l^−1^ kanamycin and stored in 20% glycerol at −80°C. The glycerol stock was inoculated into 3 ml YEB containing kanamycin and cultured at 28°C overnight at 170 rpm shaking. *Agrobacterium* cells were collected by centrifugation and suspended in the appropriate amount of MS medium containing 3% sucrose. Potato stem segments were prepared by cutting an in vitro culture and incubated with *Agrobacterium* solution. After removing the excess solution with sterilized filter paper, the segments were co-cultivated on MS medium containing 3% sucrose and 100 µM of acetosyringone for 3 days. To eliminate bacteria cells, the segments were transferred to MS medium containing 3% sucrose containing 250 mg l^−1^ carbenicillin every 2 weeks. The shoots that regenerated from the segments were isolated and cultured in plant boxes containing solidified MS medium with 3% sucrose.

### Heteroduplex mobility assay (HMA)

The regions surrounding the TALEN target sites were amplified using the primer set U1131: TCACATCTTTGGATTGTTCTCTG/U1017: TGGACCATAAATCATGCCTTC and Taq DNA polymerase (Takara Bio, Japan). The PCR products were analyzed on the MultiNA microchip electrophoresis system (MCE-202, Shimazu, Japan).

### Sequence analysis

The regions were amplified using the primer set U1131/U1017 surrounding the TALEN target sites were cloned into TOPO PCR4 Cloning Vector (Thermo Fisher Science, USA), and randomly selected colonies were used for sequencing analysis. A primer set U957: CACCATGACAGATGTTCAGGCTCC/U958: TCAATCTTCAGGCTCATCAACT was used to amplify the potential off-target site in *SSR1*, a homologue of *SSR2*.

### Steroidal glycoalkaloid (SGA) measurements of *SSR2*-edited potato plants

The leaves of in vitro cultured shoots, peels and insides of harvested tubers in green house were collected and lyophilized. α-Solanine and α-chaconine were extracted and quantified as described previously (Nakayasu et al. 2017).

### PCR analysis for transgene integration

Genomic DNA extracted and purified from leaves using Plant DNAzol (Invitrogen, USA) and diluted to 25 ng µl^−1^ or each expression vector plasmid DNA diluted to approximately 10 fg µl^−1^ were used as templates. Two µl of template was added to 20 µl of reaction solution. Each 20 µl PCR reaction solution contained 10 µl of Hot Start *Taq* 2× Master Mix (New England Biolabs, USA), and 0.2 µM forward and reverse primers. The genomic DNA from non-transgenic Sassy and plasmid DNA were used for control reactions. Primer pairs (Supplementary Tables S1, S2) were used to amplify the regions in T-DNA and backbone of the TALEN expression binary vector. Primers that amplify regions other than the target region of the *SSR2* gene were used as controls (PCR0: pY365/pY366). The PCR reaction mixture was incubated at 95°C for 30 s, followed by 34 cycles of denaturing at 95°C for 15 s, annealing at annealing temperature for 15 s, and extension at 68°C for 60 s, and final extension reaction at 68°C for 5 min. The PCR reaction mixtures were analyzed on the MultiNA microchip electrophoresis system.

### Southern hybridization analysis for transgene integration

The genomic DNA (30 µg/lane) was extracted and purified from each regenerated line using DNAzol reagent (Invitrogen, USA). Samples of the genome-edited lines, non-transgenic line (NT), non-transgenic line (NT) mixed with plasmid DNA (100 pg), and Sayaka/pYS_026-#1 in which only one copy of one set of TALEN genes is presumed to be inserted in the genome and NT Sayaka mixed with plasmid DNA (100 pg) were analyzed. Sample DNA was cleavage by restriction enzymes BamHI and HindIII. DNA probes were synthesized using the PCR DIG Probe Synthesis Kit (Sigma-Aldrich, USA) at approximately 0.5 kb within the FokI structural gene (Figure 3). CDP-Star Substrate (0.25 mM ready-To-Use, Invitrogen) was used for detection. The chemiluminescence produced was detected with a LumiCube (Liponics Corporation, Japan). Fragments of 4.1 kbp and 3.9 kbp are expected to be detected when pSuehiro108 is inserted and 3.9 kbp when pYS_026 is inserted.

### Next generation sequencing (NGS) and *k*-mer analysis for transgene integration

The genomic DNA was extracted and purified from each regenerated line using a NucleoBond® AXG Column® (Takara Bio, Japan) or DNAzol reagent (Invitrogen, USA). The sequence data were deposited in the DDBJ Sequence Read Archive (DRA) as BioProject PRJDB15512. Next generation sequencing and *k*-mer analysis for transgene integration were performed as described previously (Yasumoto and Muranaka 2023).

## Results

### Targeted genome editing in regenerated shoots on media lacking plant hormones

**Figure 1:**
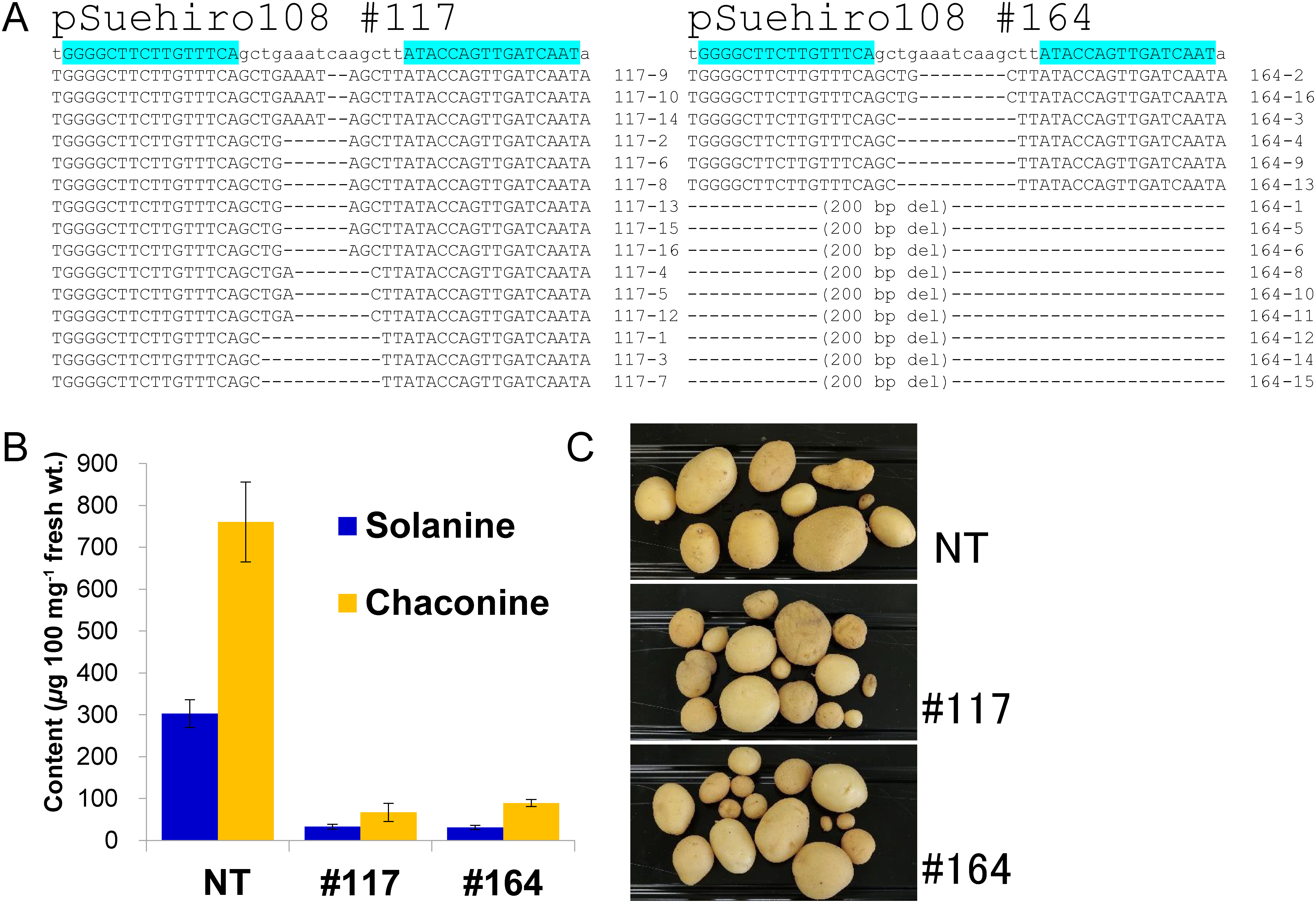
Figure 1. Completely *SSR2*-disrupted lines from “Sayaka”. (A) Sequence of target site of *SSR2* gene was amplified, cloned to E. coli, and sequenced. Pale blue indicates sequence targeted by TALEN. (B) SGA contents of in vitro shoots. Error bars indicate the standard deviation of three technical replicates. (C) Tubers harvested in green house under artificial light.

*SSR2* is the first genome-edited gene in potato and is a key gene in the steroidal glycoalkaloid (SGA) biosynthetic pathway (Sawai et al. 2014, Supplementary Figure S2). We infected potato stems with *Agrobacterium* harboring the TALEN expression vector targeting *SSR2* gene and induced shoot regeneration without a step involving selection on media lacking plant hormones. We found that no shoot buds were formed from the stem of cultivars “Sassy”, “Sayaka”, and “May Queen” treated with Agrobacterium containing the control vector pSuehiro105, i.e., vector that did not harbor the regeneration-promoting gene. In contrast, shoots were produced from stems infected by agrobacterium containing pSuehiro108 with *ipt* gene of *Agrobacterium tumefaciens* or containing a similar vector harboring and constitutively expressing one of the regeneration-promoting genes, *BBM*, *LEC2*, *ESR2*, *ESR1* or *WUS* of *Arabidopsis thaliana* (Table 1). Shoots that extended from the adventitious buds were then cultured in the same growth medium so that rooted regenerated lines were obtained. The genome of the regenerated lines was examined by heteroduplex mobility assay and PCR using primers designed using vector sequences to assess whether regenerated lines were site-specifically edited and free of vector sequences. We obtained *SSR2* gene genome-edited lines from “Sassy” from the experimental groups using the *ipt*, *BBM*, and *ESR1* genes and from “Sayaka” from the experimental groups using the *ipt* gene. None of these genome-edited lines contained T-DNA sequences (Table 1). We also obtained one genome-edited line of “Sassy” in which T-DNA sequence with the *ipt* gene was incorporated in the genome, as well as seven lines in which the T-DNA sequence with the *ipt* gene was incorporated in the genome but the genome was not edited at the target site. In these lines the internode of the regenerated shoot was narrowed and/or morphological defects such as multiple shoots were observed. Thus, it was confirmed that the *ipt* gene functioned as a negative selection marker. An amplified fragment DNA near the TALEN target sequence in the genome-edited line was then cloned into vectors and sequenced (Figure 1A, Supplementary Figures S3A, S4). The results showed genome editing in each line and that no intact sequences were found in Sassy #127, Sayaka #117, and Sayaka #164. Three different sequences were detected in Sayaka #164. Sayaka is a tetraploid variety. It has not been determined if two chromosomes have the same mutation or if another deletion mutation is larger than the primer amplification region. We then performed liquid chromatography-mass spectrometry to evaluate the SGA levels in each of these lines. Since this genome-edited region is important for the activity of *SSR2* (Yasumoto et al. 2019), deletion mutations that fit the protein translation frame are expected to eliminate *SSR2* function. As expected, these lines showed a significant reduction in SGA content compared to that of the control (Figure 1B, Supplementary Figure S3B). To confirm off-target mutations, the *SSR1* gene, the gene closest to the *SSR2* gene, was also amplified by PCR from all lines and directly sequenced; however, no mutations were identified. As previously reported (Sawai et al. 2014; Yasumoto et al. 2019), we did not observe any differences in growth habit or tubers harvested between *SSR2*-knockout lines and control plants cultivated in a greenhouse under artificial light (Figure 1C, Supplementary Figure S3C). This procedure also yielded three lines that contained an incomplete deletion of the *SSR2* gene from the “Sassy” and “Sayaka” potato cultivars (Supplementary Figures S4, S5).

**Table 1:** Table 1. Shoots and genome editing lines obtained for each regeneration-promoting gene in this experiment.

Cultivar	Regeneration-promoting gene	Explants	Regenerated shoots*	HMA positive No transgene	Complete disruptant	Efficiency (%/ Regeneration)
Sassy	—	>200	0	—	—	—
	*ipt*	427	224	2	1	0.9
	*BBM*	648	95	1	0	1.1
	*LEC2*	440	91	0	0	—
	*ESR2*	228	88	0	0	—
	*ESR1*	478	98	1	0	1.0
Sayaka	—	>200	0	—	—	—
	*ipt*	242	195	5	2	2.6
	*BBM*	266	198	0	0	—
	*ESR1*	225	115	0	0	—
	*WUS*	219	56	0	0	—
May Queen	—	>200	0	—	—	—
	*ipt*	820	282	0	0	—
	*BBM*	250	137	0	0	—
	*ESR1*	608	302	0	0	—
	*WUS*	96	13	0	0	—

* Not include the number of non-normal/multiple shoots or tissues.

### Verification of the absence of introduction of vector DNA molecules by PCR and Southern hybridization methods

**Figure 2:**
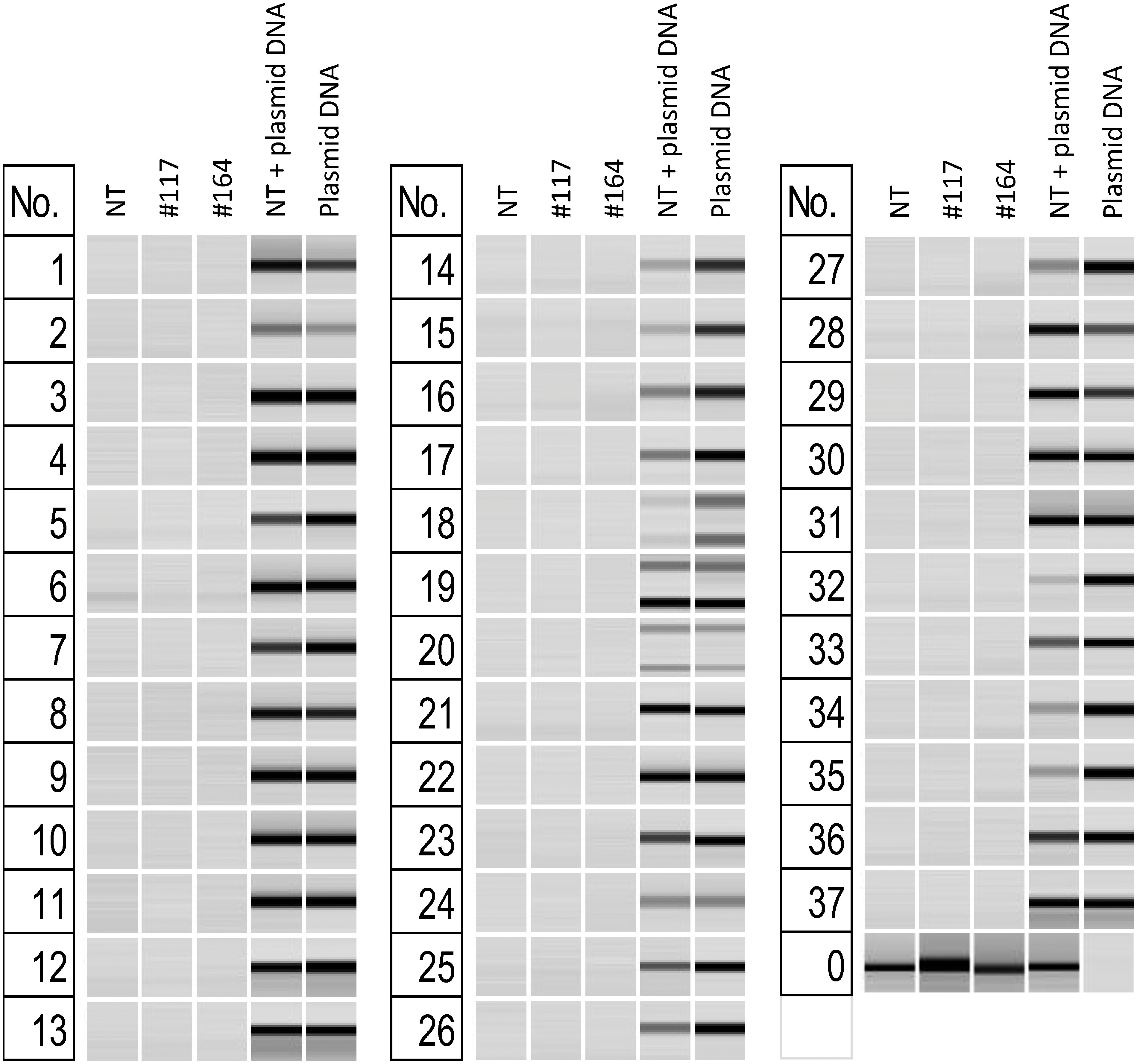
Figure 2. Verification of the absence of vector sequence using PCR. NT; non-transgenic “Sayaka”, No.; PCR set number in Supplementary Table S2. The bands that amplify in columns containing vector DNA are the sequences amplified by the PCR method. PCR18 amplifies fragments of two sizes. PCR19, PCR20 amplify fragments of three or more sizes and are representative of some of them.

During Agrobacterium transformation, in addition to the T-DNA of the binary vector, the insertion of the backbone part of the vector into the genome has been reported in rare cases. Therefore, we also designed primers for all parts of the vector used, including the backbone. To assess T-DNA integration in genome-edited regenerated plants, we performed genomic PCR using 37 primer pairs for transgenes (Supplementary Table S1, Supplementary Figure S6). The endogenous *SSR1* gene was also amplified by PCR from all lines to check the quality and quantity of the template genomic DNA. We used plasmid DNA and plasmid DNA along with DNA from the non-transgenic “Sayaka” line as positive controls. For the two lines Sayaka #117 and #164, we did not observe any integration into the genome for any sequences from any region of vector (Figure 2).

We conducted a Southern hybridization analysis on Sayaka #117 and #164 and used plasmid DNA plus DNA from non-transgenic “Sayaka”, as well as DNA obtained from a previously acquired transgenic line, as controls. The results of the Southern hybridization validation protocol, in which vector regions were used as probes, confirmed that no genome integration was detected for Sayaka #117 and #164 (Supplementary Figures S7, S8).

### Confirmation of the absence of introduction of foreign DNA molecules by NGS sequencing and *k*-mer evaluation

**Figure 3:**
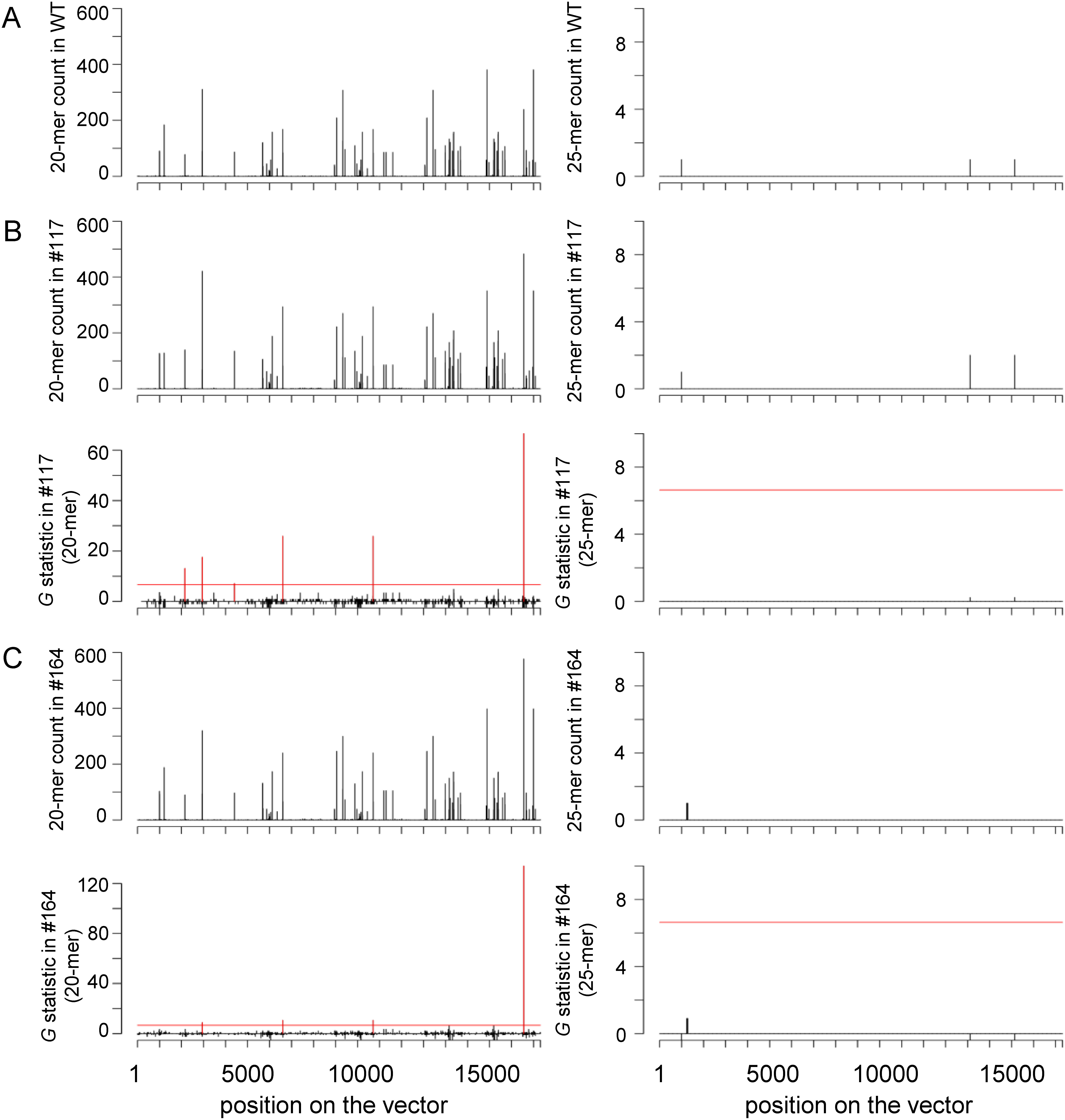
Figure 3. Detection of identical *k*-mers between the genome-edited potato genome and vector sequences. Data obtained from the wild-type (WT)(A) and selected genome-edited potato lines #117 (B), and #164 (C) are shown for *k*=20 (left) and 25 (right). The results of the counts and *G*-statistic values (against wild-type) at each position in the pSuehiro108 vector sequence used in genome-editing are shown in vertical plots. The red horizontal line corresponds to the 1% significance level (*G*-values greater than 6.634) and the vertical plot exceeding this line are shown in red. The *k*-mer analysis results obtained by repeated NGS experiments are summarized in Supplementary Tables S3, S4.

We obtained NGS data from genomic DNA extracted from wild-type potato and from the genome-edited potato lines Sayaka #117 and #164. In Sayaka #117 and #164, very few significant signals (or none) were detected during 20-mer analyses. We did not detect any significant peaks in 25-mer analyses from either line (Figure 3). NGS data acquisition and *k*-mer analysis were repeated to test whether the detected peaks were false positives. The results of all *k*-mer analyses herein are summarized in Supplementary Tables S3, S4. In the genome-edited lines, no peaks detected were common to both experiments (Supplementary Table S5). These results show that in Sayaka #117 and #164 there was no evidence of genome integration for any regions of the vector used.

## Discussion

**Figure 4:**
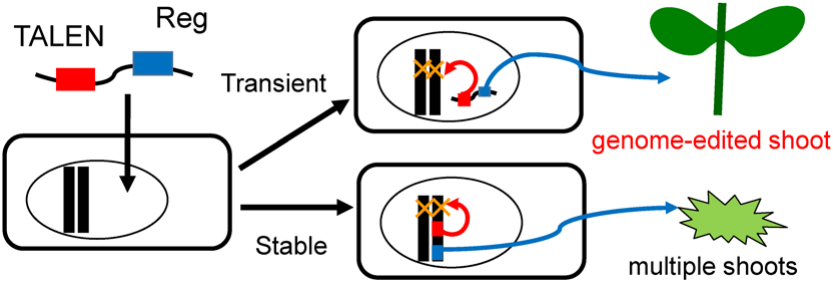
Figure 4. Schematic explanation for positive selection using transient expression of regeneration-promoting (Reg) gene.

Genome-edited plants produced by stable transformants must be treated as genetically modified organisms. The generation of null segregants, i.e., plants that harbor target mutations without transgenes, by crossing with mutants overcome this problem for seed-propagated crops, as they have a homozygous genome. However, vegetatively propagated crops, such as potato, have completely different agricultural traits from parental lines. Genome editing is possible via the transient expression of Agrobacterium, which we had previously termed “agrobacterial mutagenesis” (Yasumoto et al. 2020). The agrobacterial mutagenesis without selection is inefficient. Similarly, Chen et al. (2018) performed genome editing using *Agrobacterium*-mediated transient CRISPR-Cas9 expression in tobacco, and reported about several percent mutation rate for all regenerated shoots. They proposed to overcome the low efficiency of mutation acquisition by pooling 42 candidates and detecting a mutant among them using high-resolution melt analysis of PCR products containing genome-edited region. The efficiency of genome editing can be increased by using the genome-editing event as a selection index when the result is advantageous for survival, although most genome-editing events offer little survival advantage. Therefore, we developed a method that combined positive selection of the process of regeneration and simultaneous editing of the genome. If this method is efficient, we expect that genome-edited lines will be enriched among those that have undergone regeneration. Conveniently, regeneration-promoting genes, when inserted into the genome and constitutively expressed, induce excessive regeneration and cause morphological abnormalities such as multiple shoot formation. Thus, excluding the lines in which these genes are incorporated into the genome is possible based on plant morphology (Figure 4).

Among various regeneration-promoting genes, *ipt* was the most efficient. However, the efficiency of genome editing using *ipt* gene was less than or equal to that of the agrobacterial mutagenesis without any selection (Yasumoto et al. 2020). This may be attributed to its promotion of regeneration by mediating cytokinin synthesis. Cytokinin promotes regeneration not only in the synthesized cell but also in surrounding cells, indicating that regeneration can occur even outside the cell where the gene was transiently expressed. The efficiency of this system would therefore be improved if transcription factors that directly contribute to regeneration could be used as regeneration-promoting genes.

Using this method, we obtained genome-edited lines in the cultivars “Sassy” and “Sayaka” in which the *SSR2* gene was completely disrupted. Both cultivars are known to be easily regenerated by plant hormones. The Platinum TALEN tool used for genome editing can introduce mutations with extremely high efficiency (Yasumoto et al. 2019). We believe that the combination of these two characteristics, high efficiency of variety regeneration and high capability of genome editing tools, is suitable to perform our proposed method. “May Queen”, an older variety with a large market share in Japan, is also easily regenerated by phytohormones, but this method did not yield genome-edited potatoes. There appears to be a difference in the ease of genome editing among cultivars.

Regulations for the use of genome-edited organisms in Japan has started since 2019 with procedures including pre-submission consultation and notification to the authority of the competent minister. They are necessary to confirm that no foreign genes were introduced during the gene-editing procedure. We confirmed that the two lines #117 and #164 obtained from “Sayaka” did not contain any foreign genes and made the stipulated notification for field tests. We used PCR to detect insertions of T-DNA segments in genome-edited potatoes. We chose PCR for its high sensitivity. However, assessing the complete T-DNA region is difficult as this depends on the primers used. We performed detection of foreign DNA segments using next-generation DNA sequencing and *k*-mer analysis. When used together, these protocols may help to easily identify the presence of transgenes in the genomes of genome-edited crops. For commercial cultivation of genome-edited crops, pre-consultation for the risk assessment in the point of view of biodiversity and submission of the report to the Ministry of Agriculture, Forestry and Fisheries are required. For commercial distribution of products of genome-edited crops as food, similar procedure of consultation of assessment for the food safety and submission of the report to the Ministry of Health, Labour and Welfare are indispensable (Kondo and Taguchi 2022). Apart from those, our *SSR2* deficient potato lines are still in research stage, a similar pre-submission consultation and notification to the competent minister, MEXT permits conducting field tests using limited and regulated field facilities. We consulted MEXT for conducting the evaluation of the biological/agronomical traits under field cultivation for further research on the effects of disrupting the *SSR2* gene and finally submitted the experimental plan report with risk assessment for biodiversity on April 5, 2021. Documents related to the notification are available on the MEXT website under the “Life Science Plaza/Safety Initiatives in Life Sciences” topic. Potato, the fourth-largest food crop worldwide by production volume, can become green due to exposure to light. The green cortex and sprouts contain large amounts of SGAs such as solanine. These components cause foul taste in small amounts and toxicity in large amounts. Elimination of toxic compounds from potato is a major problem, and technical solutions are being developed to resolve this problem. From 2021, we have started field trials of two genome-edited lines with greatly reduced glycoalkaloids in Japan.

Agrobacterial mutagenesis is a useful in creating non-transgenic mutant plants without the need to segregate out transgenes by sexual reproduction, but improving the efficiency is a future challenge. Use of transiently expressed regeneration-promoting gene should be applicable to economically important and heterozygous elite crops that are more difficult to regenerate.

## Abbreviations

MEXT: the Ministry of Education, Culture, Sports, Science and Technology
TALEN: transcription activator-like effector nuclease

## Description of Supplementary Files

Supplementary Figure S1. Binary vectors in this study

Supplementary Figure S2. *SSR2* key gene in the SGA biosynthetic pathway

Supplementary Figure S3. Completely *SSR2*-disrupted lines from “Sassy”

Supplementary Figure S4. Incompletely *SSR2*-disrupted lines from “Sassy”

Supplementary Figure S5. Incompletely *SSR2*-disrupted lines from “Sayaka”

Supplementary Figure S6. Amplified regions used for PCR method

Supplementary Figure S7. Probe regions used for Southern hybridization method

Supplementary Figure S8. Verification of the absence of vector sequence using the Southern hybridization

Supplementary Table S1. Primers used to verify the absence of introduction of vector DNA molecules by PCR

Supplementary Table S2. Annealing temperature for each primer pair

Supplementary Table S3. Results of *k*-mer analyses (*k*=20)

Supplementary Table S4. Results of *k*-mer analyses (*k*=25)

Supplementary Table S5. Sequences detected above 1% significance level in three *k*-mer analyses (*k*=20, 25)
