# Supplementary material for: Integrated gene-free potato genome editing using transient transcription activator-like effector nucleases and regeneration-promoting gene expression by Agrobacterium infection
Source: Plant Biotechnol (Tokyo). 2023 Sep 25;40(3):211–8. doi: 10.5511/plantbiotechnology.23.0530a (PMC10901161; doi:10.5511/plantbiotechnology.23.0530a)
Supplement: Supplementary Data [file plantbiotechnology-40-3-23.0530a-s001.pdf]

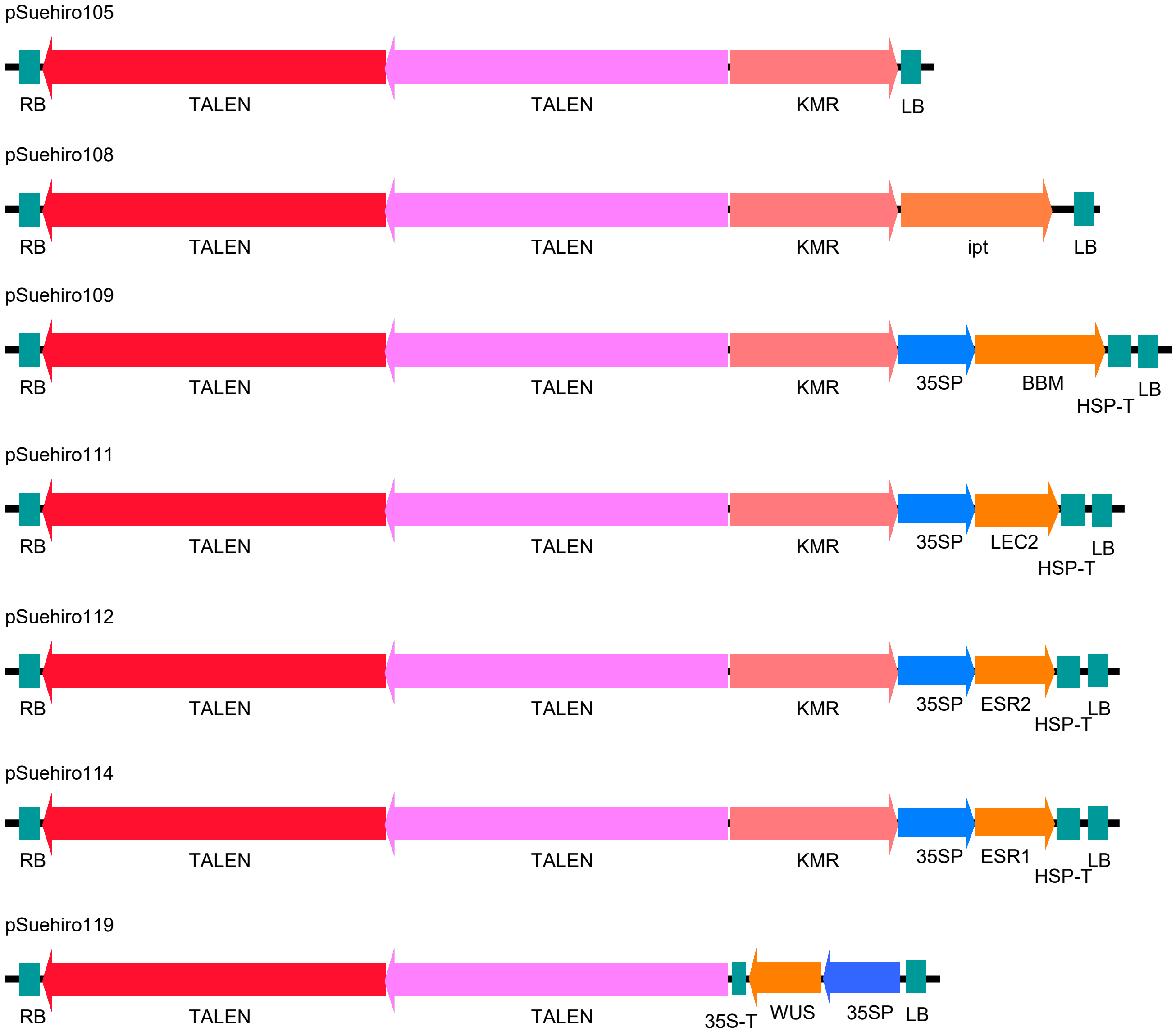

Supplementary Figure S1. Binary vectors in this study.
The structure between RB and LB was shown. RB: Right border, LB: Left border, TALEN: TALEN expression cassette, KMR: Kanamycin resistant gene expression cassette, 35SP: Cauliflower mosaic virus 35S promoter with *Arabidopsis ADH* 5' noncoding sequence, 35S-T: Cauliflower mosaic virus 35S terminator, HSP-T: *Arabidopsis* heat shock protein terminator.

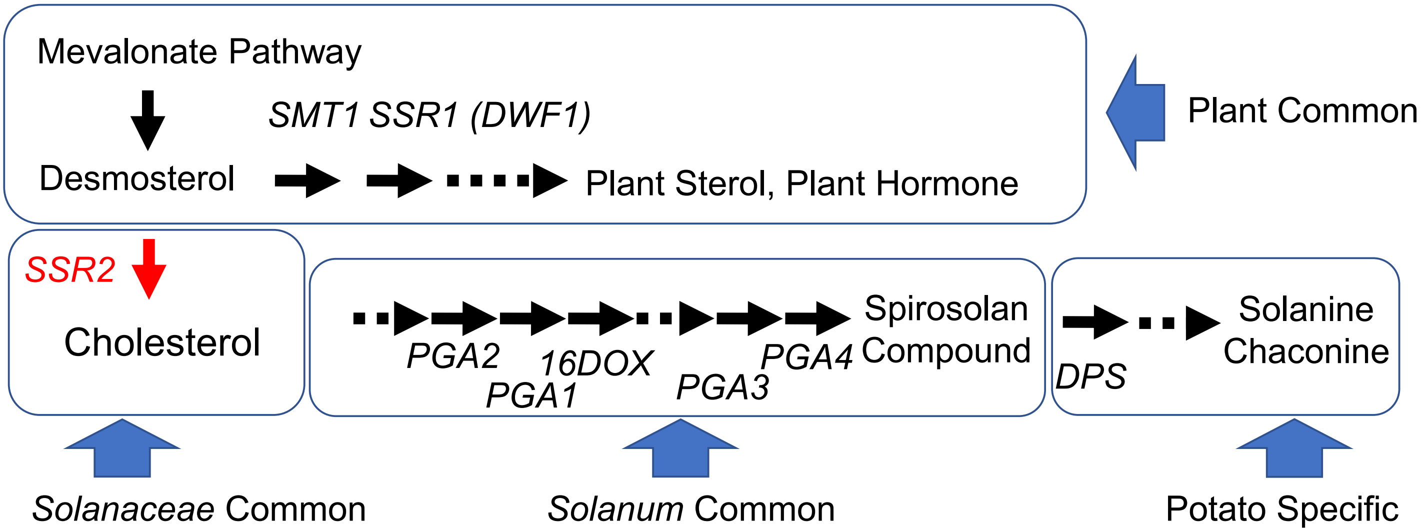

Supplementary Figure S2. *SSR2* key gene in the SGA biosynthetic pathway.
PGA1, *PGA2* (Umemoto et al. 2016), *16DOX* (Nakayasu et al. 2017), *PGA3/GAME4* (Umemoto and Sasaki 2013, Itkin et al. 2013), *PGA4* (Nakayasu et al. 2021), *DPS* (Akiyama et al. 2021). genes have been identified.

Akiyama R, Watanabe B, Nakayasu M, Lee HJ, Kato J, Umemoto N, Muranaka T, Saito K, Sugimoto Y, Mizutani M (2021) The biosynthetic pathway of potato solanidanes diverged from that of spirosolanes due to evolution of a dioxygenase. *Nat Commun* 12: 1300

Nakayasu M, Umemoto N, Ohyama K, Fujimoto Y, Lee HJ, Watanabe B, Muranaka T, Saito K, Sugimoto Y, Mizutani M (2017) A Dioxygenase Catalyzes Steroid 16alpha-Hydroxylation in Steroidal Glycoalkaloid Biosynthesis. *Plant Physiol* 175: 120-133

Nakayasu M, Umemoto N, Akiyama R, Ohyama K, Lee HJ, Miyachi H, Watanabe B, Muranaka T, Saito K, Sugimoto Y et al. (2021) Characterization of C-26 aminotransferase, indispensable for steroidal glycoalkaloid biosynthesis. *Plant J* 108: 81-92

Itkin M, Heinig U, Tzfadia O, Bhide AJ, Shinde B, Cardenas PD, Bocobza SE, Unger T, Malitsky S, Finkers R et al. (2013) Biosynthesis of antinutritional alkaloids in solanaceous crops is mediated by clustered genes. *Science* 341: 175-179

Umemoto, N, Sasaki, K (2013). Protein having glycoalkaloid biosynthetic enzyme activity and gene encoding the same. US Patent Application No. 20130167271 A1

Umemoto N, Nakayasu M, Ohyama K, Yotsu-Yamashita M, Mizutani M, Seki H, Saito K, Muranaka T (2016) Two Cytochrome P450 Monooxygenases Catalyze Early Hydroxylation Steps in the Potato Steroid Glycoalkaloid Biosynthetic Pathway. *Plant Physiol* 171: 2458-2467

A

```
tGGGGCTTCTTGTTTCAgctgaaatcaagcttATACCAGTTGATCAATa
TGGGGCTTCTTGTTTCAGCTG---------TTATACCAGTTGATCAATA
TGGGGCTTCTTGTTTCAGCTG---------TTATACCGGTTGATCAATA
TGGGGCTTCTTGTTTCAGCTG---------TTATACCGGTTGATCAATA
TGGGGCTTCTTGTTTCAGCTG---------TTATACCGGTTGATCAATA
TGGGGCTTCTTGTTTCAGCTG---------TTATACCGGTTGATCAATA
TGGGGCTTCTTGTTTCAGCTG---------TTATACCGGTTGATCAATA
TGGGGCTTCTTGTTTCAGCT------AA-CTTATACCAGTTGATCAATA
TGGGGCTTCTTGTTTCAGCT------AA-CTTATACCAGTTGATCAATA
TGGGGCTTCTTGTTTCAGCT------AA-CTTATACCAGTTGATCAATA
TGGGGCTTCTTGTTTCAGCTG-----AAGCTTATACCAGTTGATCAATA
TGGGGCTTCTTGTTTCAGCTG-----AAGCTTATACCAGTTGATCAATA
TGGGGCTTCTTGTTTCAGC-----------TTATACCAGTTGATCAATA
TGGGGCTTCTTGTTTCAGC-----------TTATACCAGTTGATCAATA
TGGGGCTTCTTGTTTCAGC-----------TTATACCAGTTGATCAATA
TGGGGCTTCTTGTTTCAGC-----------TTATACCAGTTGATCAATA
```

B

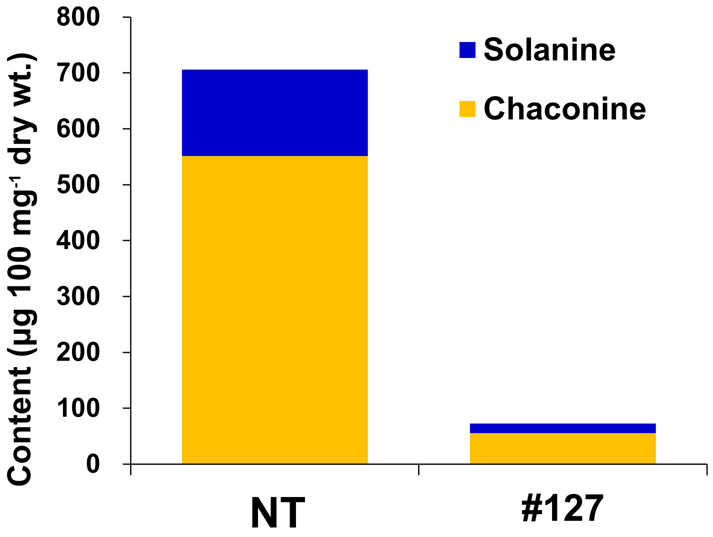

C

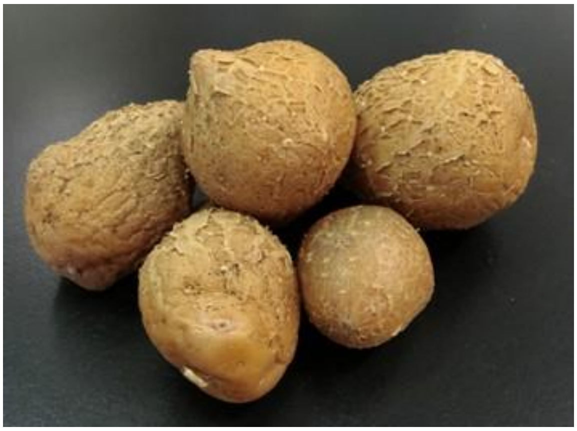

Supplementary Figure S3. Completely SSR2-disrupted lines from "Sassy".
A. Sequence of target site of *SSR2* gene was amplified, cloned to E.coli, and sequenced. Pale blue indicates sequence targeted by TALEN. In line #127, 5-11 bp deletions were observed. B. SGA contents of In vitro shoots. C. Tubers harvested in green house under artificial light.

```
pSuehiro108 #292
TGGGGCTTCTTGTTTCAgctgaaatcaagcttATACCAGTTGATCAATA
TGGGGCTTCTTGTTTCAGCTGAAATCAAGCTTATACCAGTTGATCAATA
TGGGGCTTCTTGTTTCAGCTGAAATCAAGCTTATACCAGTTGATCAATA
TGGGGCTTCTTGTTTCAGCTGAAATCAAGCTTATACCAGTTGATCAATA
TGGGGCTTCTTGTTTCAGCTGAAATCAAGCTTATACCAGTTGATCAATA
TGGGGCTTCTTGTTTCAGCTGAAATCAAGCTTATACCAGTTGATCAATA
TGGGGCTTCTTGTTTCAGCTGAAATCAAGCTTATACCAGTTGATCAATA
TGGGGCTTCTTGTTTCAGCTGAAATCAAGCTTATACCAGTTGATCAATA
TGGGGCTTCTTGTTTCAGCTGAAATCAAGCTTATACCAGTTGATCAATA
TGGGGCTTCTTGTTTCAGCTGAAATCAAGCTTATACCAGTTGATCAATA
TGGGGCTTCTTGTTTCAGCTGAAATCAAGCTTATACCAGTTGATCAATA
TGGGGCTTCTTGTTTCAGCTGAAATCAAGCTTATACCAGTTGATCAATA
TGGGGCTTCTTGTTTCAGCTGAAA----GCTTATACCAGTTGATCAATA
------------(142 bp del)-------------------------
------------(142 bp del)-------------------------
------------(142 bp del)-------------------------


pSuehiro109 #210
TGGGGCTTCTTGTTTCAgctgaaatcaagcttATACCAGTTGATCAATA
TGGGGCTTCTTGTTTCAGCTGAAATCAAGCTTATACCAGTTGATCAATA
TGGGGCTTCTTGTTTCAGCTGAAATCAAGCTTATACCAGTTGATCAATA
TGGGGCTTCTTGTTTCAGCTGAAATCAAGCTTATACCAGTTGATCAATA
TGGGGCTTCTTGTTTCAGCTGAAATCAAGCTTATACCAGTTGATCAATA
TGGGGCTTCTTGTTTCAGCTGAAATCAAGCTTATACCAGTTGATCAATA
TGGGGCTTCTTGTTTCAGCTGAAAT-AAGCTTATACCAGTTGATCAATA
TGGGGCTTCTTGTTTCAGCTGAAAT-AAGCTTATACCAGTTGATCAATA
TGGGGCTTCTTGTTTCAGCTGAAAT-AAGCTTATACCAGTTGATCAATA
TGGGGCTTCTTGTTTCAGCTGAAAT-AAGCTTATACCAGTTGATCAATA
TGGGGCTTCTTGTTTCAGCTGAAAT-AAGCTTATACCAGTTGATCAATA
-----------------(75 bp del)-------------------TA
-----------------(75 bp del)-------------------TA
-----------------(75 bp del)-------------------TA

pSuehiro114 #106
TGGGGCTTCTTGTTTCAgctgaaatcaagcttATACCAGTTGATCAATA
TGGGGCTTCTTGTTTCAGCTGAAATCAAGCTTATACCAGTTGATCAATA
TGGGGCTTCTTGTTTCAGCTGAAATCAAGCTTATACCAGTTGATCAATA
TGGGGCTTCTTGTTTCAGCTGAAATCAAGCTTATACCAGTTGATCAATA
TGGGGCTTCTTGTTTCAGCTGAAATCAAGCTTATACCAGTTGATCAATA
TGGGGCTTCTTGTTTCAGCTGAAATCAAGCTTATACCAGTTGATCAATA
TGGGGCTTCTTGTTTCAGCTGAAATCAAGCTTATACCAGTTGATCAATA
TGGGGCTTCTTGTTTCAGCTGAAATCAAGCTTATACCAGTTGATCAATA
TGGGGCTTCTTGTTTCAGCTGAAATCAAGCTTATACCAGTTGATCAATA
TGGGGCTTCTTGTTTCAGCTGAAATCAAGCTTATACCAGTTGATCAATA
TGGGGCTTCTTGTTTCAGCTGAAATCAAGCTTATACCAGTTGATCAATA
TGGGGCTTCTTGTTTCAGCTGAAATCAAGCTTATACCAGTTGATCAATA
TGGGGCTTCTTGTTTCAGCTGAAA---AGCTTATACCAGTTGATCAATA
TGGGGCTTCTTGTTTCAGCTGAAA---AGCTTATACCAGTTGATCAATA
TGGGGCTTCTTGTTTCAGCTGAAA---AGCTTATACCAGTTGATCAATA
TGGGGCTTCTTGTTTCAGCTGAAA---AGCTTATACCAGTTGATCAATA
TGGGGCTTCTTGTTTCAGCTGAAA---AGCTTATACCAGTTGATCAATA
```

Supplementary Figure S4. Incompletely SSR2-disrupted lines from "Sassy".
Sequence of target site of SSR2 gene was amplified, cloned to *E.coli*, and sequenced. Pale blue indicates sequence targeted by TALEN.

```
pSuehiro108 #63
tGGGGCTTCTTGTTTCAgctgaaatcaagcttATACCAGTTGATCAATa
TGGGGCTTCTTGTTTCAGCTGAAATCAAGCTTATACCAGTTGATCAATA
TGGGGCTTCTTGTTTCAGCTGAAATCAAGCTTATACCAGTTGATCAATA
TGGGGCTTCTTGTTTCAGCTGAAATCAAGCTTATACCAGTTGATCAATA
TGGGGCTTCTTGTTTCAGCTGAAATCAAGCTTATACCAGTTGATCAATA
TGGGGCTTCTTGTTTCAGCTGAAATCAAGCTTATACCAGTTGATCAATA
TGGGGCTTCTTGTTTCAGCTGAAATCAAGCTTATACCAGTTGATCAATA
TGGGGCTTCTTGTTTCAGCTGAAATCAAGCTTATACCAGTTGATCAATA
TGGGGCTTCTTGTTTCAGCTGAAATCAAGCTTATACCAGTTGATCAATA
TGGGGCTTCTTGTTTCAGCTGAAATCAAGCTTATACCAGTTGATCAATA
TGGGGCTTCTTGTTTCAGCTGAAATCAAGCTTATACCAGTTGATCAATA
TGGGGCTTCTTGTTTCAGCTGAAATCAAGCTTATACCAGTTGATCAATA
TGGGGCTTCTTGTTTCAGCTGAAATCAAGCTTATACCAGTTGATCAATA
TGGGGCTTCTTGTTTCAGCTGAAATCAAGCTTATACCAGTTGATCAATA
TGGGGCTTCTTGTTTCAGCTGAAATCAAGCTTATACCAGTTGATCAATA
------------(177 bp del)-------------------------
------------(199 bp del)-------------------------
```

```
pSuehiro108 #276
tGGGGCTTCTTGTTTCAgctgaaatcaagcttATACCAGTTGATCAATa
TGGGGCTTCTTGTTTCAGCTGAAATCAAGCTTATACCAGTTGATCAATA
TGGGGCTTCTTGTTTCAGCTGAAATCAAGCTTATACCAGTTGATCAATA
TGGGGCTTCTTGTTTCAGCTGAAATCAAGCTTATACCAGTTGATCAATA
TGGGGCTTCTTGTTTCAGCTGAAATCAAGCTTATACCAGTTGATCAATA
TGGGGCTTCTTGTTTCAGCTGAAATCAAGCTTATACCAGTTGATCAATA
TGGGGCTTCTTGTTTCAGCTGAAAT--AGCTTATACCAGTTGATCAATA
TGGGGCTTCTTGTTTCAGCTGAAAT--AGCTTATACCAGTTGATCAATA
TGGGGCTTCTTGTTTCAGCTGAAAT--AGCTTATACCAGTTGATCAATA
TGGGGCTTCTTGTTTC------------GCTTATACCAGTTGATCAATA
TGGGGCTTCTTGTTTC------------GCTTATACCAGTTGATCAATA
TGGGGCTTCTTGTTTC------------GCTTATACCAGTTGATCAATA
TGGGGCTTCTTGTTTC------------GCTTATACCAGTTGATCAATA
TGGGGCTTCTTGTTTC------------GCTTATACCAGTTGATCAATA
TGGGGCTTCTTGTTTC------------GCTTATACCAGTTGATCAATA
TGGGGCTTCTTGTTTC------------GCTTATACCAGTTGATCAATA
```

Supplementary Figure S5. Incompletely SSR2-disrupted lines from "Sayaka".
Sequence of target site of SSR2 gene was amplified, cloned to *E.coli*, and sequenced. Pale blue indicates sequence targeted by TALEN. For pSuehiro108 #61, which was HMA positive, sequencing did not identify any clones with deletions.

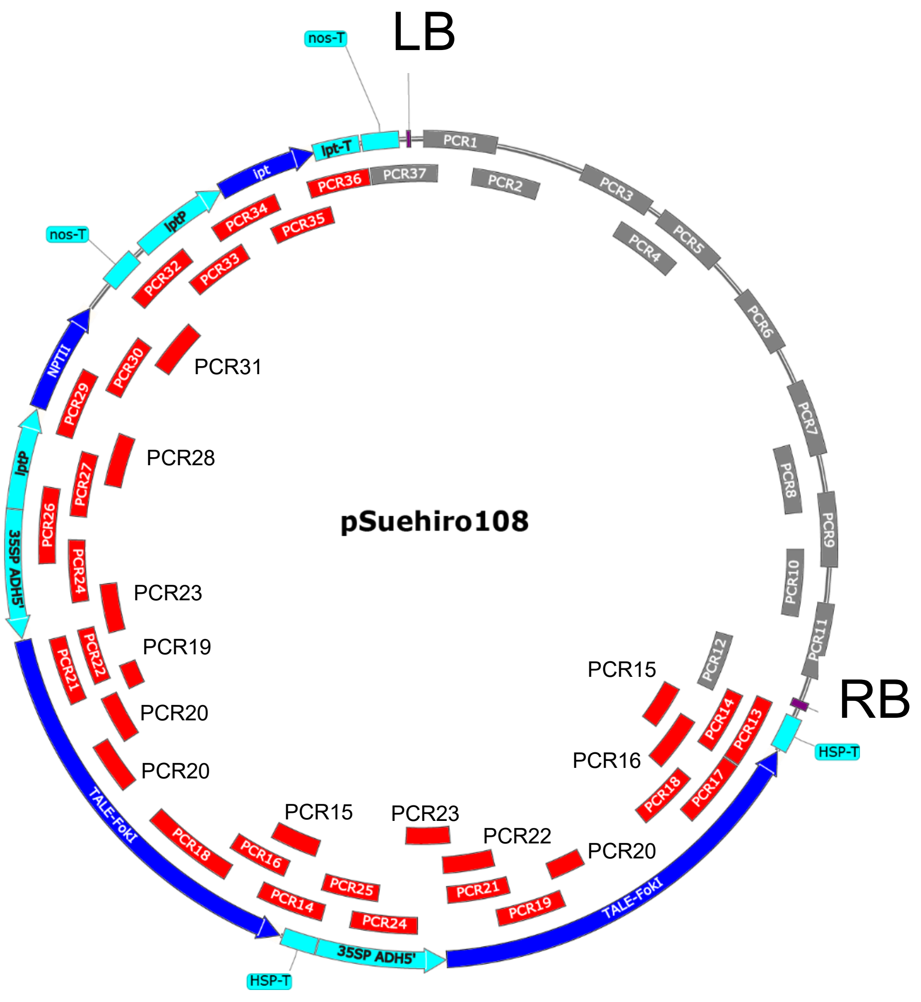

Supplementary Figure S6. Amplified regions used for PCR method.
Blue arrow, pale blue arrow , and pale box indicates gene, promoter, and terminator, respectively. Red and grey box indicates region in T-DNA and backbone of vector.

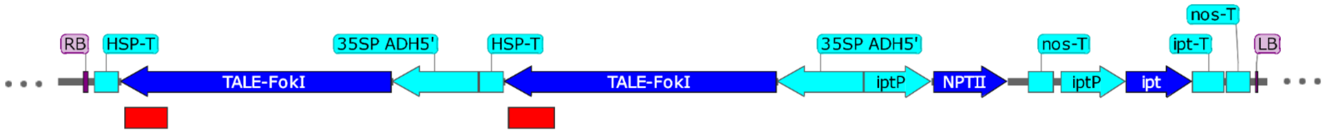

Supplementary Figure S7. Probe regions used for Southern hybridization method. Blue arrow, pale blue arrow , and pale box indicates gene, promoter, and terminator, respectively. Red box indicates Probe region.

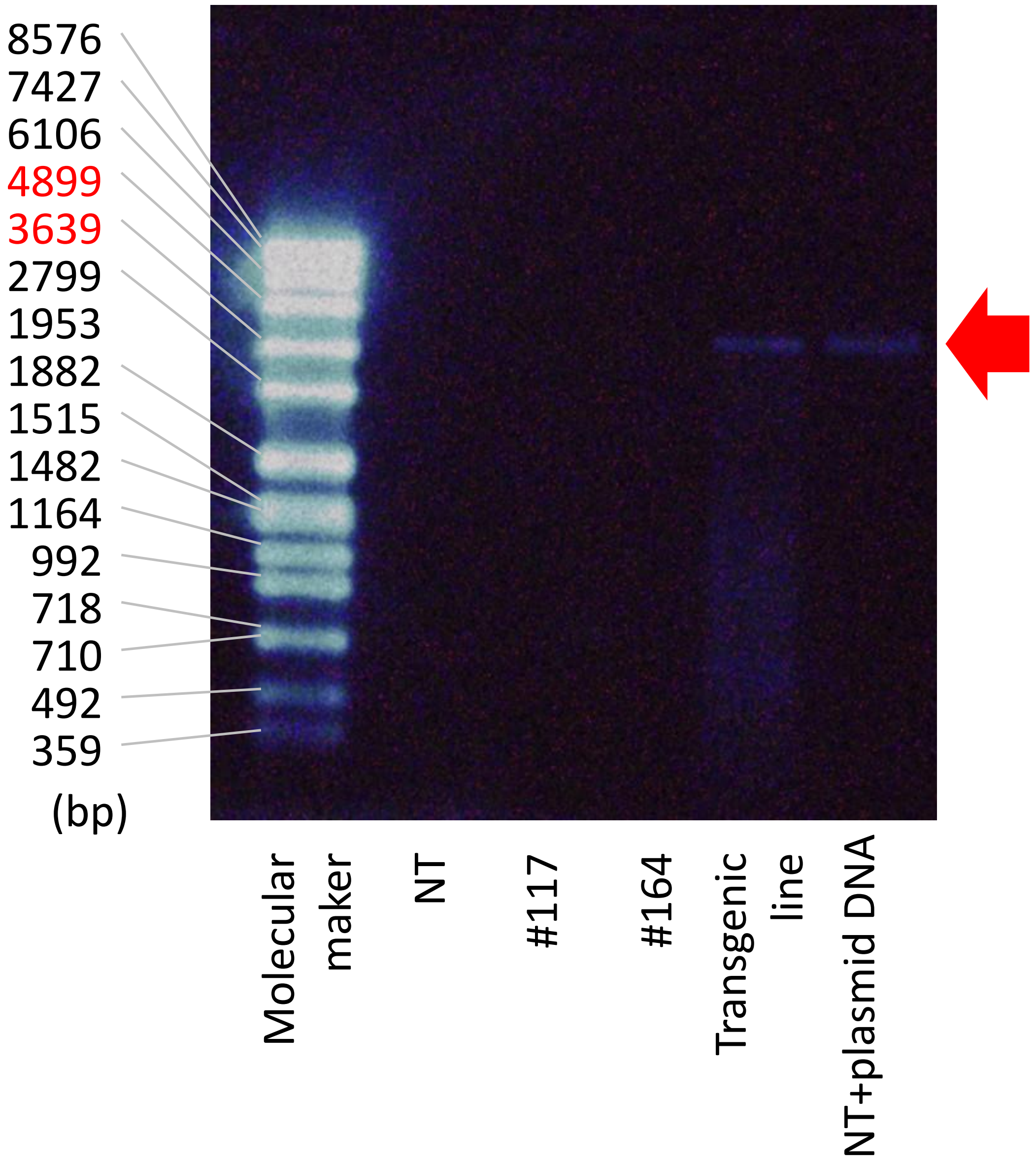

Supplementary Figure S8. Verification of the absence of vector sequence using the Southern hybridization.

Molecular Marker: DNA Molecular Weight Marker VII. DIG-labeled 5 µL (Sigma-Aldrich), NT: Sayaka non-transformed 30 µg of total DNA, #117: Sayaka pSuehiro108#117 30 µg of total DNA , #164: Sayaka pSuehiro108#164 30 µg of total DNA , Transgenic line: Sayaka Transformant pYS_026-#1 30 µg of total DNA

Supplementary Table S1. Primers used to verify the absence of introduction of vector DNA molecules by PCR.

Name	Sequence(5' to 3')
pY708/302_F1	GTTGCCATGTTTTACGGCAGTGAG
pY709/302_R500	GCTGTCCGTCATTTTTAAATATAGGTTTTC
pY711/302_R900	TTTTCCGTCTTTAAAAAATCATACAGCTCG
pY712/302_F800	ACTTACTGAATAACGATCTGGCCGA
pY748/302_F1600	TTCTCAAGATCAGAAGTACTATTCCAGTATGG
pY749/302_R2100	TGGAACGTCTTCTTTTTCCACGATGC
pY750/302_F2000	GTGGCTCCTACAAATGCCATCATTG
pY751/302_R2500	GCATGCATTCTAGACCAACCACTTG
pY752/302_F2400	AAGAAAAAGAGAAAGGTTGCGGCC
pY753/302_R2900	ACAAGTTGGCCTGTGTCCAACTGT
pY754/302_F2800	TGGAGGCCTTGCTCACGGATG
pY755/302_R3300	CTCTGGACTGTTTCTAAGGCTTGTTT
pY756/302_F3000	AGCCTTAGAAACAGTCCAGAGATTG
pY757/302_R3400	TCGTGTGACGCGATTGCAACCACCT
pY758/302_F4500	GCAGGTAGTGGCTATTGCATCCAA
pY759/302_R5000	GCTTGTTTCTTGTTTGATTCTCTTCGAC
pY760/302_F4900	CTGTCGGATCTCCTATTGATTACGGTG
pY767/302_R10100	TGACACACCAAATATTTCATCTTCATCTTC
pY771/302_R10900	GGAGAACCTGCGTGCAATCCATC
pY774/302_F11200	CTGCCGAGAAAGTATCCATCATGG
pY775/302_R11700	GTCGCTTGGTCGGTCATTTCGAA
pY776/302_F11600	CTTTACGGTATCGCCGCTCCCGAT
pY777/302_R12100	CGCGTATTAAATGTATAATTGCGGGACTC
pY778/pS108_F1	TGTGGATACCTCGCGGAAAACT
pY779/pS108_R500	TAGCGGGCCGGGAGGGTTCGAGA
pY780/pS108_F400	GCCACCGCTAACCTGTCTTTTAAC
pY781/pS108_R900	AATCTTACCTATCACCTCAAATGGTTCG
pY784/pS108_F1200	GAAACCCAGGACAATAACCTTATAGCTTG
pY785/pS108_R1700	GCCTCAACACGATTTTACGTCACT
pY786/pS108_F1600	GCCCCACTGTTCGTCCATTTC
pY787/pS108_R2100	ACGAACTCCAATTCACTGTTCCTTGC
pY788/pS108_R3100	ATCTTGCCCTGCACGAATACCA
pY791/pS108_F3400	GCGAAACCTTCCAGTCCGTCGGCT
pY792/pS108_R3900	ATAGGGGTGCGCTTCGCGTACTC
pY793/pS108_F3800	AACAAGGTCATTTTCCACGTCAACAA
pY794/pS108_R4300	GGTGGAATCCGATCCGCACAT
pY795/pS108_F4200	GGGAGAAGTACCGCAAGCTGT
pY796/pS108_R4700	GGCTTTTTCTCCTCGTGCTCGTA
pY797/pS108_F4600	TAAGGCTCAGATTCGACGGCTTG
pY798/pS108_R5100	CGGCAGAGATGAACACGACCAT
pY799/pS108_F5000	CATCCTCGGCGCACTTAATATTTCG
pY800/pS108_R5500	GGCTGAGAAAGCCCAGTAAGGAA
pY801/pS108_F5400	CCTACAGGAACCAATGTTCTCGGC
pY802/pS108_R5900	GTTTTGTTGGATCTCTTCTGCAGCA
pY803/pS108_F5800	AATGGACTAGCGGTCGGAATTCG
pY804/pS108_R6300	TGAATGGTGGAAAGTCTATCCATCTTCAG
pY805/pS108_F17900	TTAAGATTGAATCCTGTTGCCGGTCT
pY806/pS108_R100	AGCCTGCCCCTCATCTGTCAAC
pY818/pS108_F5800	CATATTCCATAGTCCATACCATAGCAC
pY819/pS108_R6300	CAAACAAGAAACAAGCATATCAACCCTA
pY820/pS108_F6200	TCTTGTAAGCTGAGCTTTGTAGTTTCC
pY821/pS108_R6700	GATGCAGTGAAAAAGGGATTGCC
pY822/pS108_F6600	AAGTTCAGATTTCTTCTCTTCCAATTCA
pY823/pS108_R7100	CGGAGGCAAACAAGCCTTAGAAAC
pY824/pS108_F8400	GTGCTCCCGTGAGCGCATTGC
pY825/pS108_R8900	GGCCGCTGACTACAAGGATGA
pY826/pS108_F9500	ACTCTGTATGAACTGTTCGCCAGT
pY827/pS108_R10000	GTTTTGTTGGATCTCTTCTGCAGCA
pY828/pS108_R14200	TGAAATAACGGCGCCATTTGAACA
pY829/pS108_F14000	CAGGCATGCAAGCTTACAAGTATTG
pY830/pS108_R14500	GGCTGTCTCCTTGAAAGCAATTTGTC
pY831/pS108_F14300	GTCAATTGTGAAATAGCCGCCCT
pY832/pS108_F14600	ATCGAGGAAGAGAATATAACAGCCTC
pY833/pS108_R15100	CAGGTAGCCGGATCAAGCGTAT
pY834/pS108_R16300	CTTAAATGGCCCTATAGCTGGGC
pY835/pS108_F16200	GTTCAAATGGCGCCGTTATTTCA
pY836/pS108_R16700	ATTAGACGTAGATCCATCGGTCTTG
pY837/pS108_F16600	CCTAAAGCCACTTGCTCTTCAAGGA
pY838/pS108_R17100	AACATCTGCTTAACTCTGGTCTTGG
pY839/pS108_F17000	GGATCTATCTCGTTGCTCAGGTG
pY840/pS108_R17500	TTCAAACCACTTGCCTCTATCACAAC
pY841/pS108_F17400	CGTTTGAAGGACCACCATTTCG
pY842/pS108_R17900	TGATAATCATCGCAAGACCGGCA
pY365/1682/check-for	TGTTCTCTGACACTGTTGTAGCACT
pY366/1683/check-rev	TCGAAGCATACATACCGGTCATCAT

Supplementary Table S2. Annealing temperature for each primer pair.

No.	Primer	Tm(°C)
1	pY778/pY779	56
2	pY780/pY781	53
3	pY784/pY785	54
4	pY786/pY787	56
5	pY708/pY709	51
6	pY712/pY788	55
7	pY791/pY792	61
8	pY793/pY794	54
9	pY795/pY796	57
10	pY797/pY798	57
11	pY799/pY800	55
12	pY801/pY802	55
13	pY803/pY804	55

No.	Primer	Tm(°C)
14	pY818/pY819	52
15	pY760/pY767	52
16	pY820/pY821	54
17	pY758/pY759	53
18	pY822/pY823	51
19	pY754/pY755	54
20	pY756/pY757	53
21	pY824/pY825	57
22	pY752/pY753	55
23	pY750/pY751	56
24	pY748/pY749	54
25	pY826/pY827	55
26	pY826/pY828	55

No.	Primer	Tm(°C)
27	pY829/pY830	54
28	pY831/pY771	55
29	pY832/pY833	53
30	pY774/pY775	55
31	pY776/pY777	55
32	pY805/pY834	55
33	pY835/pY836	53
34	pY837/pY838	54
35	pY839/pY840	54
36	pY841/pY842	54
37	pY805/pY806	55
0	pY365/pY366	55
